# Distribution characteristics of SARS-CoV-2 IgM/IgG in false-positive results detected by chemiluminescent immunoassay

**DOI:** 10.1515/biol-2022-0512

**Published:** 2022-11-14

**Authors:** Yan Lei, Xiaolan Lu, Daiyong Mou, Qin Du, Guangrong Wang, Qiang Wang

**Affiliations:** Department of Clinical Laboratory, Affiliated Hospital of North Sichuan Medical College, Nanchong, 637000, Sichuan, China; Department of Laboratory Medicine, North Sichuan Medical College; Translational Medicine Research Center, North Sichuan Medical College, Nanchong, 637000, Sichuan, China; Department of Clinical Laboratory, Nanchong Central Hospital, Nanchong, 637000, Sichuan, China

**Keywords:** SARS-CoV-2, COVID-19, antibody, chemiluminescent immunoassay, immunoglobulin M, immunoglobulin G, false positive

## Abstract

There have been several false-positive results in the antibody detection of COVID-19. This study aimed to analyze the distribution characteristics of severe acute respiratory syndrome coronavirus 2 (SARS-CoV-2) immunoglobulin M (IgM) and immunoglobulin G (IgG) in false-positive results using chemiluminescent immunoassay. The characteristics of false-positive results in SARS-CoV-2 IgM and IgG tests were analyzed. The false-positive proportion of single SARS-CoV-2 IgM-positive results was 95.88%, which was higher than those of single SARS-CoV-2 IgG-positive results (71.05%; *p* < 0.001) and SARS-CoV-2 IgM- and IgG-positive results (39.39%; *p* < 0.001). The S/CO ratios of SARS-CoV-2 IgM and IgG in false-positive results ranged from 1.0 to 50.0. The false-positive probability of SARS-CoV-2 IgM in the ratios of specimen signals to the cutoff value (S/CO) range (1.0–3.0) was 95.06% (77/81), and the probability of false-positive results of SARS-CoV-2 IgG in the S/CO range (1.0–2.0) was 85.71% (24/28). Dynamic monitoring showed that the S/CO values of IgM in false-positive results decreased or remained unchanged, whereas the S/CO values of IgG in false-positive results decreased. The possibility of false-positive single SARS-CoV-2 IgM-positive and single SARS-CoV-2 IgG-positive results was high. As the value of S/CO ratios decreased, the probability of false-positives consequently increased, especially among the single SARS-CoV-2 IgM-positive results.

## 1 Introduction

The coronavirus disease 2019 (COVID-19) pandemic, caused by severe acute respiratory syndrome coronavirus 2 (SARS-CoV-2), has had a catastrophic effect on the world’s population, becoming the most consequential global health crisis since the influenza pandemic of 1918 [1,2,3]. A range of nucleic acid-based and antigen/antibody-based tests are available to detect SARS-CoV-2 infection [4]. While nucleic acid-based or antigen detection tests are used for diagnostic purposes, antibody detection tests may be used to assess exposure to the virus or conduct serosurveillance of populations [4,5]. In the early stages, the detection of novel coronavirus-specific antibodies in individuals who are not vaccinated with the novel coronavirus vaccine can be used as a reference for diagnosis [6].

Currently, serological testing products for COVID-19 include chemiluminescent immunoassay (CLIA), gold immunochromatography assay, enzyme-linked immunosorbent assay (ELISA), electrochemiluminescence assay (ECLIA), lateral flow immunoassay (LFI) and so on [7,8,9,10,11]. CLIA is a central laboratory-based method that can provide high-throughput testing using serum/plasma. SARS-CoV-2 immunoglobulin M (IgM) and immunoglobulin G (IgG) antibodies with CLIA assays have high sensitivity and specificity, but false-positive results occur regularly [12,13]. Our previously published work showed that false-positive SARS-CoV-2 IgM results could be caused by a moderate to a high concentration of rheumatoid factor IgM in the patient’s serum [14]. There have been some interferences resulting in false-positive results of SARS-CoV-2 serological tests, such as certain pathological factors, biological factors, other endogenous interference factors, other viral infections, and cross-reactions [15,16]

Despite the wide spread of SARS-CoV-2, most areas around the world still have an overall low seroprevalence in the unvaccinated population [17,18]. The predictive value of a positive test will be lower in individuals with a low background risk of infection, especially in low-prevalence settings [19]. Interpretation of a positive test in a patient with a low pretest probability should be performed with caution, as false-positives could lead to potential harm to the patient.

Based on the earlier, there is a need to distinguish false-positive results from true-positive results in serological tests. Moreover, there are limited published data about false-positive results in SARS-CoV-2 IgM and IgG testing. Therefore, we analyzed the characteristics of false-positive results in SARS-CoV-2 IgM and IgG testing using the CLIA method. We further propose an alternative strategy for COVID-19 false-positive serological tests, enabling the best use of serological tests in immunological and seroprevalence studies of SARS-CoV-2.

## 2 Materials and methods

### 2.1 Participants and case definition

We conducted this study in two academic hospitals in Nanchong (population of 7.15 million), located in northeastern Sichuan Province in China. In this study, the cases were from two cohorts of non-SARS-CoV-2 and SARS-CoV-2 patients collected in Nanchong. The first cohort included 14,673 patients admitted to the Affiliated Hospital of North Sichuan Medical College and Nanchong Central Hospital, Nanchong, China from March 2020 to September 2020. All these patients in the first cohort were unvaccinated and had no epidemiological risk of contracting SARS-CoV-2 infection, including recent travel history to a country with a community outbreak of COVID-19 or contact with symptomatic people who had traveled back from a country with a community outbreak of COVID-19. In the second cohort, we recruited only 35 COVID-19 patients from March 2020 to December 2020 in Nanchong, where a total of 39 confirmed COVID-19 cases were reported. The confirmed number was maintained and did not increase throughout 2021.

A confirmed COVID-19 case was defined based on the Diagnosis and Treatment Protocol for COVID-19 (Tentative 8th Edition) released by the National Health Commission of the People’s Republic of China [6]. Briefly, a confirmed case had to meet three criteria: (1) fever and/or respiratory symptoms; (2) abnormal lung imaging findings; and (3) a positive SARS-CoV-2 nucleic acid test result.


**Informed consent:** Informed consent has been obtained from all individuals included in this study.
**Ethical approval:** The research related to human use has been complied with all the relevant national regulations, institutional policies and in accordance with the tenets of the Helsinki Declaration, and has been approved by the ethics committee of the Affiliated Hospital of North Sichuan Medical College (NO. 2020ER008-1).

### 2.2 Sample collection

Paired throat swabs and blood samples were taken from each patient. Residual blood samples were analyzed after the requested standard-of-care laboratory testing. In a subset of participants, blood samples from SARS-CoV-2 IgM false-positive cases and true-positive cases were obtained 30 days after the first positive serological tests.

### 2.3 Measurement of SARS-CoV-2 IgM and IgG

SARS-CoV-2 IgM and IgG tests were both performed for these cases. SARS-CoV-2 IgM and IgG serum levels were measured using an automatic CLIA system (Bioscience Biotechnology Co., Ltd.) with reagents, including SARS-CoV-2 antibody (IgM and IgG) detection kits (Bioscience Biotechnology Co., Ltd., lot number for IgM: G202002415, IgG: G202002414). Briefly, IgG and IgM antibody detection was developed based on CLIA, which uses recombinant antigens containing the receptor-binding domain (RBD) of the SARS-CoV-2 spike protein, acting as the conjugated antigen. The antibody levels were expressed by the chemiluminescence signal. The commercially available assay used in this study has been evaluated to be sufficiently sensitive and specific for detecting SARS-CoV-2 IgM and IgG in clinical specimens [20]. According to the manufacturer’s instructions and standard operating procedures, daily maintenance was performed before sample testing, and both internal quality control and sample testing were carried out afterward.

All the samples that showed initial positive antibody test results were retested and confirmed by another SARS-CoV-2 antibody (IgM and IgG) detection kit (Sichuan Orienter Biotechnology Co., Ltd.) using an automatic CLIA system.

### 2.4 SARS-CoV-2 RNA testing

Nasal and pharyngeal swabs (Baso, Zhuhai, Guangdong, China) were collected from all enrolled patients. According to the manufacturer’s instructions, RNA was extracted using a nucleic acid automated extraction system (Zhijiang, Shanghai, China). A commercial multiplex real-time PCR kit (Maccura2019-nCoV Assay, Chengdu, Sichuan, China) was then used to detect the ORF1ab, N, and E genes of SARS-CoV-2. The results were considered positive when two or three genes were identified.

### 2.5 Result judgment

SARS-CoV-2 IgM and IgG results detected by CLIA were given in the form of the ratios of specimen signals to the cutoff values (S/CO), which were nonreactive (negative) if the S/CO ratio was < 1.0 and reactive (positive) if the S/CO ratio was ≥ 1.0 by the manufacturer. Moreover, a “false-positive” case was defined as a positive antibody result from a non-COVID-19-infected patient. A “true-positive” case was defined as a positive antibody result from a COVID-19 patient.

### 2.6 Statistical analysis

Measurement data were displayed as mean ± standard deviation (SD), and count data are expressed as percentages. The chi-square test was used to compare enumeration data. All statistical analyses were performed using SPSS version 23.0 (SPSS Co., Inc., Chicago, IL), and statistical significance was defined as *p* < 0.05, as determined using two-tailed tests.

## 3 Results

### 3.1 Comparison of the proportion of false-positives in single SARS-CoV-2 IgM-, single SARS-CoV-2 IgG-, and SARS-CoV-2 IgM- & IgG- positive results

The IgM and IgG of 14,708 patients were tested for SARS-CoV-2 in this study, excluding cases with duplicated patients. A total of 168 (1.19%) patients with positive results (S/CO ratio ≥ 1.0) were included for further analysis. Among these 168 cases, 35 (20.83%) were defined as “true positives,” and the remaining 133 (79.17%) were defined as “false-positives.”

Ninety-seven of 168 (57.74%) cases had single SARS-CoV-2 IgM-positive results: 4/97 (4.12%) were true positives, and 93/97 (95.88%) were false-positives. Thirty-eight of 168 (22.62%) cases had single SARS-CoV-2 IgG-positive results: 11/38 (28.95%) were true positives, and 27/38 (71.05%) were false-positives. Thirty-three of 168 (19.64%) cases had SARS-CoV-2 IgM- and IgG-positive results: 20/33 (60.61%) were true positives, and 13/33 (39.39%) were false positives. The false-positive proportion of single SARS-CoV-2 IgM-positive results (95.88%) was significantly higher than those of single SARS-CoV-2 IgG-positive results (71.05%; *p* < 0.001) and SARS-CoV-2 IgM- & IgG-positive results (39.39%; *p* < 0.001 [Table 1]).

**Table 1:** Comparison of the proportion of true-positives and false-positives in single SARS-CoV-2 IgM-, single SARS-CoV-2 IgG-, and SARS-CoV-2 IgM- and IgG- positive results

	*n*	SARS-CoV-2	
		True-positive (*n* [%])	Fasle-positive (*n* [%])
Single IgM (+)	97	4 (4.12%)	93 (95.88%)
Single IgG (+)	38	11 (28.95%)	27 (71.05%)^*^
IgM & IgG (+)	33	20 (60.61%)	13 (39.39%)^*#^
*χ* ^2^		49.589	
*p*-Value		<0.0001	

Note: *p < 0.001, when compared with the single SARS-CoV-2 IgM positive. ^#^ *p* < 0.01, when compared with the single SARS-CoV-2 IgG positive.

### 3.2 Comparison of the false-positive proportion of single SARS-CoV-2 IgM-, single SARS-CoV-2 IgG-, and SARS-CoV-2 IgM- and IgG-positive results in different sexes and ages

In this study, 69 of 133 (51.88%) false-positives were observed among males: 43/69 (62.32%) had a single SARS-CoV-2 IgM-positive result, 18/69 (26.09%) had a single SARS-CoV-2 IgG-positive result, and 8/69 (11.59%) had SARS-CoV-2 IgM- & IgG-positive results. A total of 64 of 133 (48.12%) false-positives were observed among females: 50/64 (78.13%) had a single SARS-CoV-2 IgM-positive result, 9/64 (14.06%) had a single SARS-CoV-2 IgG-positive result, and 5/64 (7.81%) had SARS-CoV-2 IgM- and IgG-positive results. Based on these results, no significant difference was found in the false-positive proportion of the three result patterns between sexes (*p* > 0.05; Table 2). Similarly, no significant difference was found in the false-positive rates of the three result patterns in different age groups (*p* > 0.05; Table 3).

**Table 2:** Comparison of the false-positives proportion of single SARS-CoV-2 IgM-, single SARS-CoV-2 IgG-, and SARS-CoV-2 IgM- and IgG- positive results in different sexes

	Single IgM (+)	Single IgG (+)	IgM and IgG (+)
Male	62.32% (43/69)	26.09% (18/69)	11.59% (8/69)
Female	78.13% (50/64)	14.06% (9/64)	7.81% (5/64)
χ^2^	3.229	2.967	0.538
*p*-Value	0.072	0.085	0.463

**Table 3:** Comparison of the false-positives proportion of single SARS-CoV-2 IgM-, single SARS-CoV-2 IgG-, and SARS-CoV-2 IgM- and IgG- positive results in different ages

Age (years)	Single IgM (+)	Single IgG (+)	IgM and IgG (+)
≤20	54.55% (12/22)	40.91% (9/22)	4.55% (1/22)
21∼	69.23% (9/13)	15.38% (2/13)	15.38% (2/13)
31∼	72.00% (18/25)	20.00% (5/25)	8.00% (2/25)
41∼	68.42% (13/19)	10.53% (2/19)	21.05% (4/19)
51∼	65.22% (15/23)	17.39% (4/23)	17.39% (4/23)
61∼	90.91% (10/11)	9.09% (1/11)	0.00% (0/11)
>70	80.00% (16/20)	20.00% (4/20)	0.00% (0/20)
*χ* ^2^/Fisher	6.060	6.802	7.912
*p*-Value	0.426	0.327	0.169

### 3.3 Distribution of SARS-CoV-2 IgM S/CO values in false-positive and positive results

**Figure 1:**
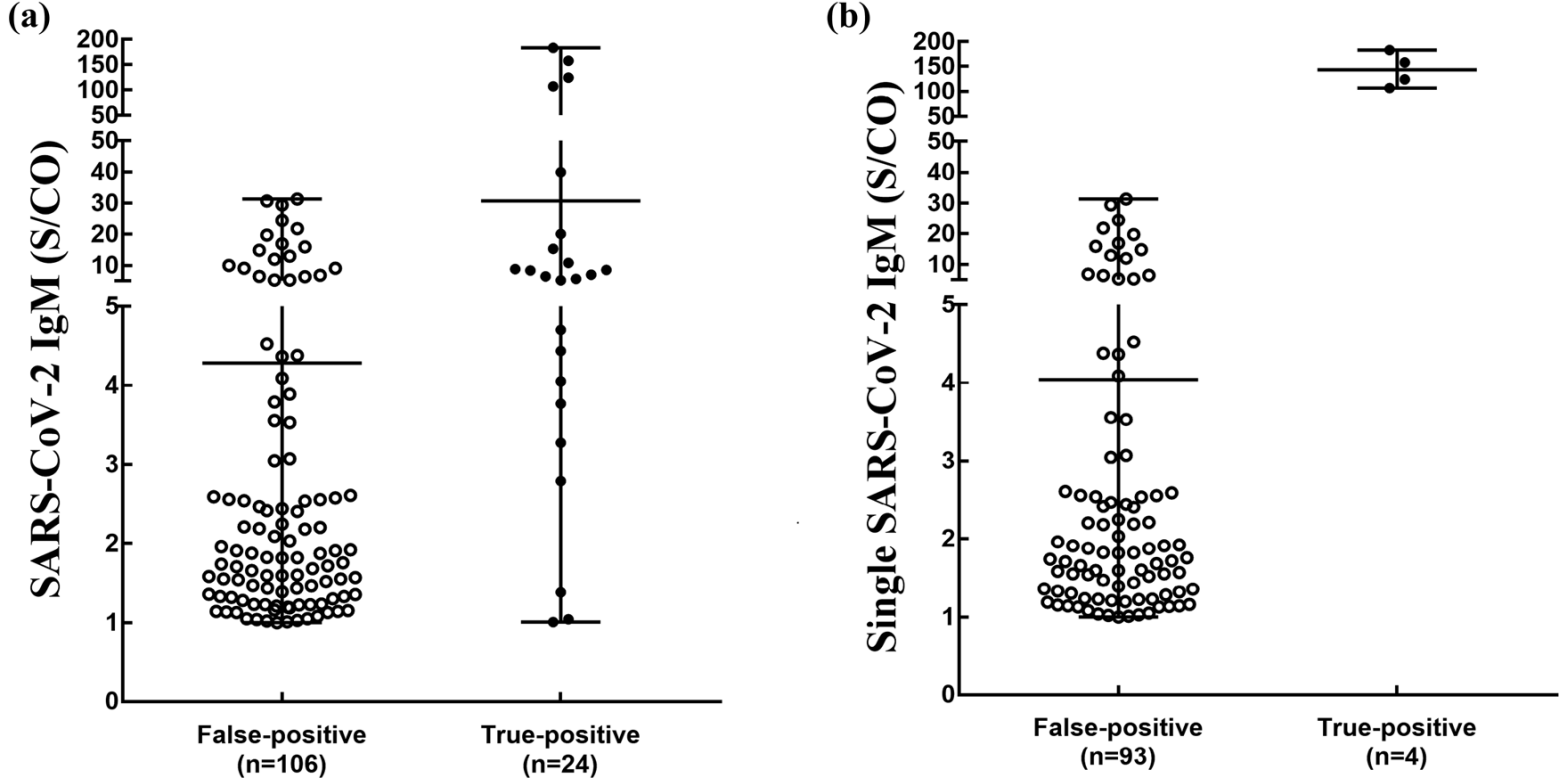
Distribution of SARS-CoV-2 IgM S/CO results greater than or equal to 1.0. (a) Distribution of S/CO values of SARS-CoV-2 IgM in false-positive and positive results. (b) Distribution of S/CO values of single SARS-CoV-2 IgM in false-positive and positive results. S/CO = ratios of specimen signals to the cut-off values.

Among the cases in our study, 130 had SARS-CoV-2 IgM S/CO values greater than or equal to 1.0. A total of 97/130 (74.62%) cases had single SARS-CoV-2 IgM-positive results. Figure 1 shows the distribution of SARS-CoV-2 IgM S/CO values in the false-positive and true-positive results. The S/CO values of IgM false-positives ranged from 1.0 to 32.0, with most of them concentrated between 1.0 and 3.0 (Figure 1a and b). The S/CO values of the SARS-CoV-2 IgM true-positive cases ranged from 1.0 to 180.0, with most of them between 3.0 and 10.0 (Figure 1a), whereas the S/CO values of single SARS-CoV-2 IgM true-positive cases were all more than 100.0 (Figure 1b). The overlapping range of S/CO values for SARS-CoV-2 IgM false-positive and true-positive cases was mainly from 1.0 to 50.0 (Figure 1a). Notably, neither SARS-CoV-2 IgM nor single SARS-CoV-2 IgM in false-positive cases showed S/CO values greater than 40.0 (Figure 1a and b).

As shown in Table 4, when the S/CO values of SARS-CoV-2 IgM were in the ranges of 1.0–3.0, 3.0–5.0, 5.0–10.0, 10.0–50.0, and greater than 50.0, the probability of SARS-CoV-2 IgM false-positives was 95.06, 66.67, 50.00, 75.00, and 0.00%, respectively. Meanwhile, when the S/CO values of single SARS-CoV-2 IgM were in the ranges of 1.0–50.0 and greater than 50.0, the probability of a single SARS-CoV-2 IgM false-positive result was 100.00 and 0.00%, respectively.

**Table 4:** Probability of the false-positive results in SARS-CoV-2 IgM and single SARS-CoV-2 IgM positive results

S/CO	SARS-CoV-2 IgM		Single SARS-CoV-2 IgM	
	False-positive	True-positive	False-positive	True-positive
1.0–3.0	95.06% (77/81)^*^	4.94% (4/81)	100.00% (70/70)^#^	0.00% (0/70)
3.0–5.0	66.67% (10/15)^*^	33.33% (5/15)	100.00% (8/8)^#^	0.00% (0/8)
5.0–10.0	50.00% (7/14)^*^	50.00% (7/14)	100.00% (5/5)^#^	0.00% (0/5)
10.0–50.0	75.00% (12/16)^*^	25.00% (4/16)	100.00% (10/10)^#^	0.00% (0/10)
>50.0	0.00% (0/4)	100.00% (4/4)	0.00% (0/4)	100.00% (4/4)
Fisher	34.775		28.635	
*p*-Value	<0.0001		<0.0001	

Note: S/CO = ratios of specimen signals to the cut-off values. ^*^ *p* < 0.001, when compared with the SARS-CoV-2 IgM in ranges greater than 50.0. ^#^ *p* < 0.001, when compared with the single SARS-CoV-2 IgM in ranges greater than 50.0.

### 3.4 Distribution of SARS-CoV-2 IgG S/CO values in false-positive and positive results

**Figure 2:**
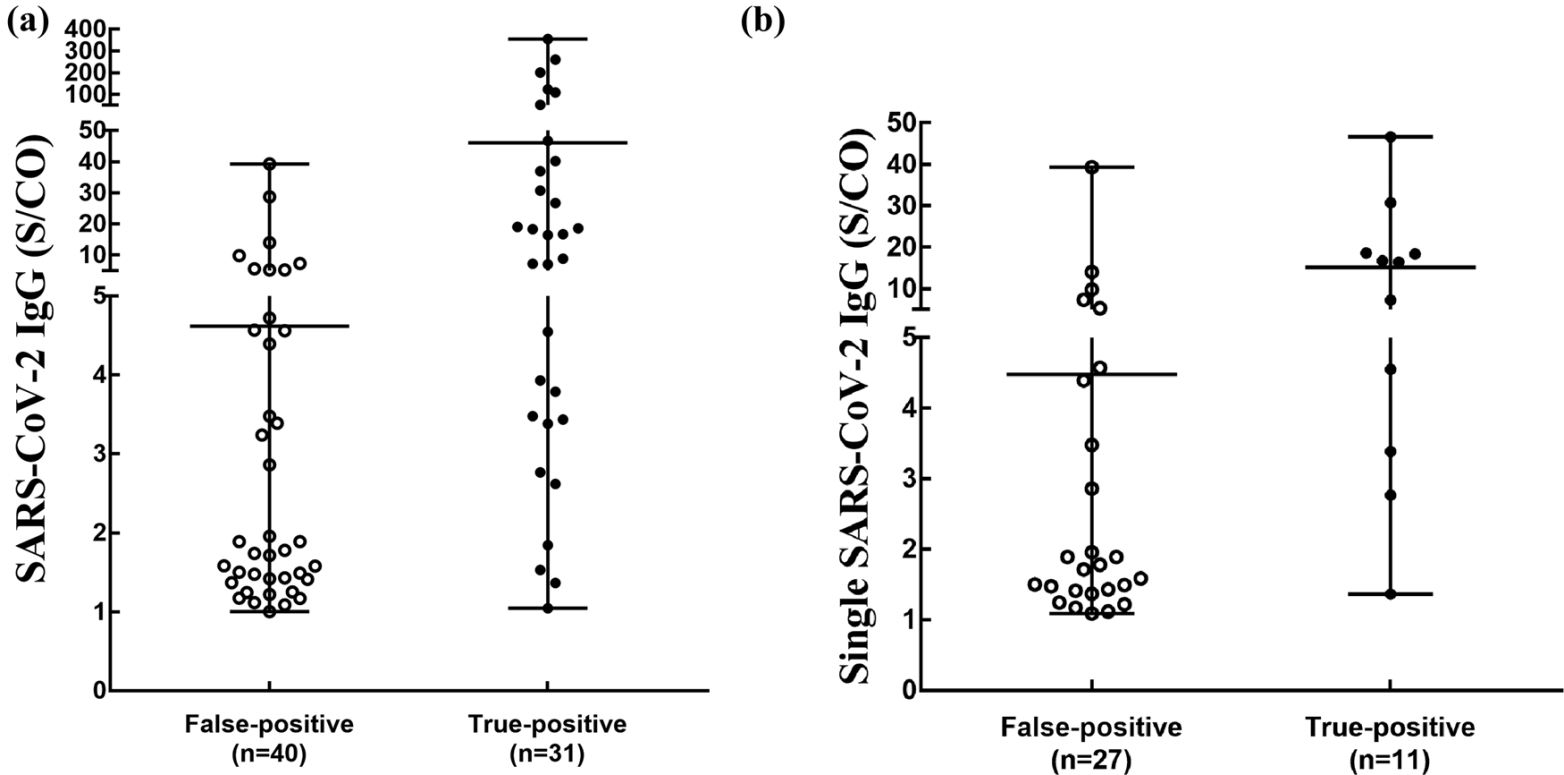
Distribution of SARS-CoV-2 IgG S/CO values greater than or equal to 1.0. (a) Distribution of S/CO values of SARS-CoV-2 IgG in false-positive and positive results. (b) Distribution of S/CO values of single SARS-CoV-2 IgG in false-positive and positive results. S/CO = ratios of specimen signals to the cut-off values.

Of the 71 cases with S/CO values of SARS-CoV-2 IgG greater than or equal to 1.0, 38/71 (53.52%) cases had single SARS-CoV-2 IgG-positive results. Figure 2 shows the distribution of SARS-CoV-2 IgG S/CO values in false-positive and true-positive results. The S/CO values of false-positives ranged from 1.0 to 40.0, with most concentrated between 1.0 and 2.0, whereas the S/CO values of true positives ranged from 1.0 to 360.0, with most above 10.0. Moreover, the overlapping range of S/CO values for SARS-CoV-2 IgG-positive and false-positive cases was mainly from 1.0 to 50.0 (Figure 2a and b). Interestingly, S/CO values above 40.0 indicated no false-positive SARS-CoV-2 IgG results (Figure 2a and b).

When the S/CO values of SARS-CoV-2 IgG were in the ranges of 1.0–2.0, 2.0–5.0, 5.0–10.0, 10.0–50.0, and greater than 50.0, the probability of SARS-CoV-2 IgG false-positives was 85.71, 50.00, 62.5, 23.08, and 0.00%, respectively. When the S/CO values of single SARS-CoV-2 IgG were in the ranges of 1.0–2.0, 2.0–5.0, 5.0–10.0, and 10.0–50.0, the probability of false-positive single SARS-CoV-2 IgG results was 94.74, 57.14, 75.00, and 25.00%, respectively (Table 5).

**Table 5:** Probability of the false-positive results in SARS-CoV-2 IgG and single SARS-CoV-2 IgG-positive results

S/CO	SARS-CoV-2 IgG		Single SARS-CoV-2 IgG	
	False-positive	Positive	False-positive	Positive
1.0–2.0	85.71% (24/28)^*&^	14.29% (4/28)	94.74% (18/19)^#^	5.26% (1/19)
2.0–5.0	50.00% (8/16)^*&^	50.00% (8/16)	57.14% (4/7)^#^	42.84% (3/7)
5.0–10.0	62.50% (5/8)^*&^	37.50% (3/8)	75.00% (3/4)^#^	25.00% (1/4)
10.0–50.0	23.08% (3/13)^*^	76.92% (10/13)	25.00% (2/8)	75.00% (6/8)
>50.0	0.00% (0/6)	100.00% (6/6)	—	—
Fisher	24.416		13.892	
*p*-Value	<0.0001		0.001	

Note: S/CO = ratios of specimen signals to the cut-off values. ^*^ *p* < 0.001, when compared with the SARS-CoV-2 IgG in ranges of greater than 50.0; ^&^ *p* < 0.01, when compared with the SARS-CoV-2 IgG in ranges of 10.0–50.0. ^#^ *p* < 0.01, when compared with the single SARS-CoV-2 IgG in ranges of 10.0–50.0.

### 3.5 Dynamic changes in SARS-CoV-2 IgM and IgG in false-positive cases

**Figure 3:**
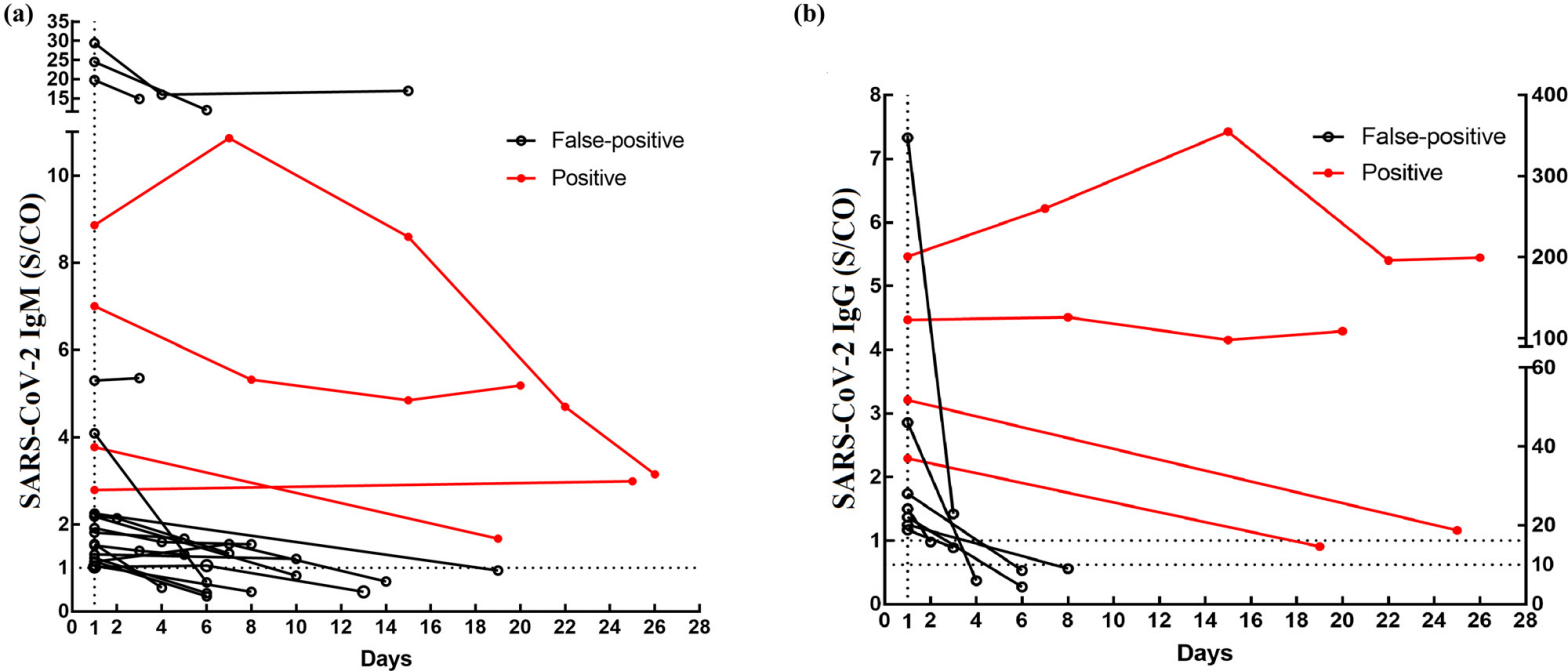
Dynamic changes in SARS-CoV-2 specific antibody in false-positive or positive results. (a) Dynamic changes of SARS-CoV-2 IgM in false-positive results. (b) Dynamic changes of SARS-CoV-2 IgG in false-positive results. The left vertical axis was suitable for the false-positive results, and the right vertical axis was suitable for the positive results. The axis “Days” represents the days after the first test.

Eighteen SARS-CoV-2 IgM false-positive cases and four true-positive cases were dynamically monitored at different time points. Of the 18 false-positive cases, 10 cases turned negative, and the remaining eight cases did not. The median time of conversion to seronegative status in the 10 cases was 9 days. Compared with the dynamic change trend of true-positive cases, the changing trend in SARS-CoV-2 IgM false-positive cases was substantially lower. On the other hand, among the eight false-positive cases that were not monitored to become seronegative, three cases showed a downward trend, and five remained unchanged (Figure 3a).

Seven SARS-CoV-2 IgG false-positive cases and four true-positive cases were dynamically monitored at different time points. Of the seven false-positive cases, six became seronegative, and only one case did not. The median time of conversion to seronegative status of the six cases was 5 days. Compared with the dynamic change trend of SARS-CoV-2 IgG in true-positive cases, the changing trend in false-positive cases was substantially lower. Notably, only one false-positive case, which was not monitored as seronegative, showed a downward trend (Figure 3b).

## 4 Discussion

During the COVID-19 pandemic, assay kits for detecting IgM and IgG antibodies against SARS-CoV-2 proteins have expanded our measures for COVID-19 detection [13,21]. Antibodies for SARS-CoV-2 are produced within the range of 5–14 days after the onset of disease symptoms [22,23]. Almost all COVID-19 patients develop detectable IgG and IgM antibodies within several weeks of symptom onset [13,21,24]. The low cost, ease of performing and interpreting, and fast turn-around time make serological tests attractive for quick diagnosis and mass screening [25]. Furthermore, detecting specific antibodies has been proven to show public health and clinical utility for pandemic monitoring and response and managing affected patients [5,26].

In our study, we used the CLIA method based on the recombinant SARS-CoV-2 S-RBD protein to detect serum IgG and IgM. Its analytical performance was successfully evaluated by Wan et al. They reported the performance verification of SARS-CoV-2 IgM (82% sensitivity and 93.85% specificity) and SARS-CoV-2 IgG (86% sensitivity and 96.92% specificity) detection kits among COVID-19 patients [20]. Due to the problems of immunological detection methods, some interferences resulted in false-positive results of SARS-CoV-2 serological tests that were inconsistent with clinical manifestations and epidemiological characteristics [14,15,16]. False-positive signals were detected in serological tests using CLIA and reported in other immunoassay analytical techniques, such as ELISA, ECLIA, and LFI [11]. Additionally, some false-positive cases likely did not result from problems with the sample, procedure, or other random factors, which was supported by obtaining repeated positive results with similar S/CO values on repeat testing (data not shown) in our study, making a transient response to antigens less likely. Although we can use electronic medical records and laboratory results, including nucleic acid-based tests, as a source to determine true anti-SARS-CoV-2 status, the procedure is labor-intensive and time-consuming. Therefore, we sought an effective strategy to solve such problems when confronted with false-positive results in SARS-CoV-2 antibody screening tests.

To elucidate these issues, we analyzed the false-positive cases of SARS-CoV-2 IgM and IgG detected using CLIA among non-SARS-CoV-2-infected patients. This study showed that the false-positive proportion of single SARS-CoV-2 IgM-positive results was 95.88%, substantially higher than those of single SARS-CoV-2 IgG-positive results (71.05%) and SARS-CoV-2 IgM- and IgG-positive results (39.39%). Other studies have also reported that most false-positive signals were detected in SARS-CoV-2 IgM assays [14,15,27]. These false-positive results of SARS-CoV-2 IgM and IgG antibodies may be caused by autoantibodies, heterophilic antibodies, and other factors [12,14,15,16]. Therefore, we concluded that the possibility of false-positive single SARS-CoV-2 IgM-positive and single SARS-CoV-2 IgG-positive results was high. The false-positive rate of IgM and IgG together is lower than when examined individually. In this study, we also observed true-positive cases of SARS-CoV-2 IgM and IgG in confirmed COVID-19 patients. The true-positive proportions of single SARS-CoV-2 IgM-, single SARS-CoV-2 IgG- and SARS-CoV-2 IgM- & IgG-positive results were 4.12, 28.95, and 60.61%, respectively. The combined detection of SARS-CoV-2 IgM and IgG antibodies is an effective tool for improving diagnostic sensitivity and specificity [12]. Thus, the combined detection of SARS-CoV-2 IgM and IgG antibodies should be given high priority in its implementation as the standard serological test in clinical and public health practice during the pandemic.

In this study, we found that the S/CO values of the IgM false-positive results were mainly between 1.0 and 3.0, whereas the S/CO values of the IgG false-positive results were mainly between 1.0 and 2.0. These results indicated that the SARS-CoV-2 IgM and IgG false-positive results detected by CLIA primarily existed in the low-value area. The false-positive results, which were low positives or low values, needed further confirmation. Therefore, the S/CO value may be a helpful indicator in differentiating false positives from true positives. Aside from the S/CO values, we also compared the false-positive proportions of single SARS-CoV-2 IgM-, single SARS-CoV-2 IgG-, and SARS-CoV-2 IgM- and IgG-positive results in different sexes and ages. Our study showed no significant false-positive proportion differences between sexes and ages.

After SARS-CoV-2 invades the human body, the time and duration of producing IgM and IgG antibodies are different. Due to the dynamic change in specific antibodies, cases with a single positive SARS-CoV-2 IgM/IgG antibody test can be determined by dynamically monitoring them over time [6]. Despite this, the question of how long the supposed antibody level can last in false-positive cases remains unknown. To investigate this, we analyzed the subsequent results of four true-positive cases and 25 false-positive cases, including 18 cases with a SARS-CoV-2 IgM-positive result and seven cases with a SARS-CoV-2 IgG-positive result, and dynamic monitoring of the serum antibody levels after the first test. The time of conversion to seronegativity in IgM false-positive cases was 4–19 days, with a median time of 9 days. Moreover, the antibody duration in the IgM false-positive cases was substantially shorter than that in COVID-19 patients with a duration of 2–6 months, as reported in other studies [28,29]. Meanwhile, the conversion time to seronegativity in IgG false-positive cases was 2–8 days, with a median seroconversion time of 5 days. Compared with previous studies, the antibody duration in the IgG false-positive cases was also substantially shorter than the duration of serum IgG antibodies in COVID-19 patients (6 months) [28,29]. For other cases observed during the monitoring period and those that did not become seronegative, IgM antibody levels showed a downward trend or remained unchanged. Similarly, IgG antibody levels in those cases also showed a downward trend. Due to the lack of blood samples collected from the false-positive cases in the later stage, the time of conversion in their antibodies remained unknown. In this study, the results suggest that the dynamic monitoring of serum antibody levels was also of practical value in distinguishing between true-positive and false-positive results.

To summarize, these findings should be comprehensively judged when confronted with positive results in SARS-CoV-2-specific antibody testing. First, it is crucial to observe the antibody pattern. If it is a single SARS-CoV-2 IgM-positive pattern, the probability of a false-positive result is higher than that of a single SARS-CoV-2 IgG-positive pattern. Moreover, if it is a SARS-CoV-2 IgM- and IgG-positive pattern, the probability of a true-positive result is higher than that of a single positive antibody result. Second, one must observe the S/CO value distribution of the antibody results as follows: (1) if the S/CO value of a single SARS-CoV-2 IgG-positive result is between 1.0 and 2.0, the probability of a false-positive is approximately 94.74% (Table 5); (2) if the S/CO value of a single SARS-CoV-2 IgM-positive result is between 1.0 and 3.0, the probability of a false-positive is approximately 100.0% (Table 4); (3) if a single SARS-CoV-2 IgM test has a positive result, and the S/CO value is very high (>20.0), the result needs to be judged in combination with the S/CO value of the SARS-CoV-2 IgG result; (3a) if the S/CO value of the SARS-CoV-2 IgG result approaches 0, there is a high possibility of a false-positive result; and (3b) if the S/CO value of the SARS-CoV-2 IgG result is close to 1.0, it is more likely to be a true-positive result. Last, antibody level changes should be observed dynamically. In cases of a single SARS-CoV-2 IgM-positive status, IgM antibody increases, and IgG antibody becomes positive (i.e., IgM- and IgG-positive status), with which we can judge if the patient truly has a SARS-CoV-2 infection. Otherwise, it is a false-positive result. On the other hand, in the dynamic monitoring of SARS-CoV-2-positive IgM and positive IgG status, the IgM antibody is the first to increase and subsequently decrease, whereas the IgG antibody titer has a 4-fold increase, with which we can judge if the patient truly has a SARS-CoV-2 infection [6]. Otherwise, these are false-positive results. Furthermore, the single SARS-CoV-2 IgG-positive status shows a rapidly decreasing trend in dynamic monitoring, which may indicate a false-positive result.

Despite the findings of our study, several limitations were noted. (1) Due to the inadequate conditions of our laboratory, we failed to determine the definite interferences or factors causing false-positive results. (2) The disease prevalence of the population is not known. The false-positive rate is highly dependent on the disease prevalence. (3) Since most of the SARS-COV-2-specific antibody detection was performed using the CLIA platform due to its high throughput, our research analysis focused only on CLIA. Thus, this study’s characteristics of false-positive results may not apply to other SARS-COV-2 antibody detection methods. Regardless, this study can still provide a reference strategy for other researchers.

## 5 Conclusion

The results of our study confirm that separate positive SARS-COV-2 IgM and IgG antibody tests would be an unconvincing result. We proposed that the possible usage of the SARS-COV-2 IgM and IgG antibody patterns, S/CO values or ranges, and dynamic changes in antibody levels are useful for screening positive antibody results. This study aimed to assist clinicians or technicians in developing timely and effective diagnostic strategies to distinguish false-positive results from true-positive results. However, regardless of the method, laboratories should develop approaches to identify analytical false-positive results wherever possible, particularly when screening low-prevalence populations.
